# Reply: Fibrosis Is Inevitable, but Heterogeneous: Time-Dependent and Biochemical Modifiers of Liver Injury After Fontan Completion

**DOI:** 10.1016/j.jacadv.2026.102762

**Published:** 2026-04-22

**Authors:** Jack Rychik

**Affiliations:** a FORWARD Clinic Program at Children’s Hospital of Philadelphia, Philadelphia, Pennsylvania, USA; b Robert & Dolores Harrington Endowed Chair in Pediatric Cardiology, Philadelphia, Pennsylvania, USA; c Perelman School of Medicine, University of Pennsylvania, Philadelphia, Pennsylvania, USA

Many thanks for Dr Shah’s insights derived from our work.^1^ We wholeheartedly agree with his points. Liver fibrosis in the Fontan circulation is indeed an expression of multiple factors in a complex formula, ultimately manifesting in a specific degree and magnitude of disease. The proposed model is one in which several physiologic stressors, as well as systems of resilience, come together in a complex formula resulting in a precise state that is unique to each individual affected. The condition is strongly influenced by the overlying foundation of altered hemodynamics of hepatic congestion present in all, yet likely modified by various additional processes still to be elucidated. Bile acid metabolism and its regulation is certainly one such candidate area to explore with possibilities for therapy.^2^ Additional work investigating fundamental impacts on the biology of organ, cellular, and metabolic functions as well as insights from “omics” studies^3^ offer the best opportunities to discover clues to modifying and ameliorating the deleterious wide-ranging effects of the Fontan circulation “syndrome.”
